# Report of a phase 1 clinical trial for safety assessment of human placental mesenchymal stem cells therapy in patients with critical limb ischemia (CLI)

**DOI:** 10.1186/s13287-023-03390-9

**Published:** 2023-07-05

**Authors:** Zeinab Shirbaghaee, Saeed Heidari Keshel, Mehdi Rasouli, Majid Valizadeh, Seyed Saeed Hashemi Nazari, Mohammad Hassani, Masoud Soleimani

**Affiliations:** 1 Department of Tissue Engineering and Applied Cell Sciences, School of Advanced Technologies in Medicine, Shahid Beheshti University of Medical Sciences, Tehran, Iran; 2 Obesity Research Center, Research Institute for Endocrine Sciences, Shahid Beheshti University of Medical Sciences, Tehran, Iran; 3 Prevention of Cardiovascular Disease Research Center, Department of Epidemiology, School of Public Health and Safety, Shahid Beheshti University of Medical Sciences, Tehran, Iran; 4 Department of Vascular and Endovascular Surgery, Ayatollah Taleghani Hospital Research Development Committee, Shahid Beheshti University of Medical Sciences, Velenjak St., Shahid Chamran Highway, Tehran, Iran; 5 Applied Cell Sciences and Hematology Department, Faculty of Medical Sciences, Tarbiat Modares University, Tehran, Iran

**Keywords:** Critical limb ischemia, Peripheral artery disease, Buerger, Placenta, Mesenchymal stem cell, Clinical trial, Cell therapy

## Abstract

**Background:**

Critical limb ischemia (CLI) is associated with increased risk of tissue loss, leading to significant morbidity and mortality. Therapeutic angiogenesis using cell-based treatments, notably mesenchymal stem cells (MSCs), is essential for enhancing blood flow to ischemic areas in subjects suffering from CLI. The objective of this study was to evaluate the feasibility of using placenta-derived mesenchymal stem cells (P-MSCs) in patients with CLI.

**Methods:**

This phase I dose-escalation study investigated P-MSCs in nine CLI patients who were enrolled into each of the two dosage groups (20 × 10^6^ and 60 × 10^6^ cells), delivered intramuscularly twice, two months apart. The incidence of treatment-related adverse events was the primary endpoint. The decrease in inflammatory cytokines, improvement in the ankle-brachial pressure index (ABI), maximum walking distance, vascular collateralization, alleviation of rest pain, healing of ulceration, and avoidance of major amputation in the target leg were the efficacy outcomes.

**Results:**

All dosages of P-MSCs, including the highest tested dose of 60 × 10^6^ cells, were well tolerated. During the 6-month follow-up period, there was a statistically significant decrease in IL-1 and IFN-γ serum levels following P-MSC treatment. The blood lymphocyte profile of participants with CLI did not significantly differ, suggesting that the injection of allogeneic cells did not cause T-cell proliferation in vivo. We found clinically substantial improvement in rest pain, ulcer healing, and maximum walking distance after P-MSC implantation. In patients with CLI, we performed minor amputations rather than major amputations. Angiography was unable to demonstrate new small vessels formation significantly.

**Conclusion:**

The observations from this phase I clinical study indicate that intramuscular administration of P-MSCs is considered safe and well tolerated and may dramatically improve physical performance and minimize inflammatory conditions in patients with CLI.

*Trial registration*: IRCT, IRCT20210221050446N1. Registered May 09, 2021.

**Supplementary Information:**

The online version contains supplementary material available at 10.1186/s13287-023-03390-9.

## Introduction

Peripheral artery disease (PAD), except for aortic and coronary involvement, includes all arterial disorders. It is mainly associated with extracranial carotids, upper extremity arteries, renal and mesenteric arteries, and lower limb arteries [1]. PAD is often defined as ischemia of the extremities caused by atherosclerotic obliterans (ASO). Another cause of PAD is vasculitis, which includes thromboangiitis obliterans (TAO) (Buerger's disease) and can result in severe limb ischemia [2]. Buerger's disease is an inflammatory disease of vessels, perivascular tissues, and nerves that is distinguished by thrombosis in the arterial vascular system. It primarily impacts young individuals who have a history of chronic tobacco consumption and can affect the lower and even upper extremities [3]. These illnesses lower life quality globally, raise healthcare expenditures, and increase mortality rates [4]. As a result, the prevalence of PAD increases with age due to persistent smoking, increased type 2 diabetes, obesity, and sedentary lifestyle [5].

Critical limb ischemia (CLI), the most severe form of PAD, results in ischemic rest pain, unhealed wounds, gangrene, and limb amputations. Endovascular revascularization or open surgery is the current standard treatment for improving blood flow to affected limbs [6]. However, 20–40% of individuals are ineligible for these treatments because of inadequate internal vascular anatomy or a high risk of surgery [7]. Moreover, no drug has been approved by the Food and Drug Administration (FDA) to treat patients with CLI. Managing the disease's complications through wound care, pain control, and ultimately, amputation is occasionally the last resort for these individuals with no other options who are not eligible for surgery. According to estimates, the mortality rate of these patients is 30% and the risk of amputation is 40% [8]. Given therapeutic limits and a high mortality rate, the quality of life of individuals with CLI is comparable to that of individuals with terminal-stage cancer. Since CLI is a socioeconomic hardship in communities, appropriate treatment is necessary for the patient [9]. In recent years, new and more effective treatments have evolved, such as stem cell therapy, which uses stem cells as a feasible alternative for treating these no-option patients [10–12].

Vascular regeneration processes, such as angiogenesis and arteriogenesis, occur in ischemic lesions in healthy tissues. Yet, this phenomenon does not occur in CLI [13]. As blood vessels help regenerate damaged tissue by providing oxygen and nutrients, angiogenesis-based therapies stimulate the formation of new vessels in these tissues [14]. Owing to their unique vasculogenic properties and paracrine implications, cell-based therapies are thought to be more effective than strategies focused on proteins or genes [15, 16]. Mesenchymal stem cells (MSCs) are regarded as a regenerative therapy for critical limb ischemia owing to their distinct biological features [17]. MSCs have the ability to develop into endothelial cells (ECs) and produce angiogenic factors. Therefore, they can promote tissue regeneration, improve function, induce angiogenesis, and restore blood circulation to ischemic regions [18].

In preclinical studies employing animal models of lower-extremity ischemia, autologous and allogeneic MSCs produced from various sources, including bone marrow, adipose tissue, and prenatal sources, have been beneficial [19]. Following this, many clinical trials have been conducted to demonstrate the feasibility and effectiveness of MSCs produced from multiple sources for the therapy of CLI as a consequence of positive preclinical research results [20].

MSCs improve the endurance of pre-existing blood vessels in co-culture with ECs in a dose-dependent manner [17, 21]. In order to start the early stages of angiogenesis, complex crosstalk between MSCs and ECs lowers the permeability of the EC monolayer. Several studies have demonstrated that one of MSCs' primary tasks is to secrete bioactive substances relevant to the niche where they are implanted [22]. MSCs support homeostasis and possess anti-apoptotic properties, but they also have immunomodulatory capabilities in inflammatory conditions that inhibit the development and manufacture of cytokines in effector cells. MSCs can inhibit various immune cell activities. Consequently, they can serve as sources of allogeneic stem cells for regenerative medicine without cell rejection [23].

The placenta is a highly vascular tissue that receives several angiogenesis-stimulating impulses. This feature makes it an ideal milieu for signals that promote angiogenesis. Placenta-derived mesenchymal stem cells (P-MSCs) exhibit morphological features nearly identical to those of other MSCs, which are detected in the vascular niche of the placenta [24, 25]. Lee et al. investigated the differentiation power of human P-MSC cells into smooth muscle by transplanting these cells into mouse placenta and showed that the interaction between these two types of cells plays an important role in vascularization [26]. Xie et al. conducted a preclinical study to investigate the efficacy of P-MSC cells in a mouse model of hind limb ischemia (HLI). They found that P-MSCs significantly increased the density of microvessels, increased blood perfusion at the injury site, and eliminated the symptoms of the disease compared with the control group. These cells cooperate with other cells in the degree of angiogenesis and have the ability to differentiate into endothelial cells and smooth vascular cells to increase angiogenesis in organs involved in ischemia [27].

P-MSCs are an excellent example of fetal stem cells that have been suggested as substitutes for medical applications [28]. P-MSCs can maintain long telomeres, exhibit pluripotency markers including Oct4 and SSEA-3, and undergo trilineage differentiation [29].

Similar to other MSCs, P-MSCs exhibit a limited level of HLA-A, HLA-B, and HLA-C molecules, which reduces their immunogenicity and increases their suitability for transplantation. P-MSCs cause pro-inflammatory M1 cells to transform into anti-inflammatory M2 cells. He S.et al. showed that placental mesenchymal cells (PDA-002) injected into a mouse model of HLI increased M2 anti-inflammatory macrophages in ischemic tissue. They also induce the cytokine profile and gene expression of Th2 lymphocytes. These findings indicate that the increase in angiogenesis of PDA-002 cells occurs through an immunomodulatory mechanism in which T lymphocytes play a key role in the differentiation and reprogramming of macrophages toward the M2 type [30].

Additionally, P-MSCs expressing HLA-G inhibit T cell development [31]. A comparison experiment between mononuclear cells and dendritic cells using UCB-MSCs and P-MSCs revealed that P-MSC conditioned media (CM) suppressed T cell proliferation substantially more than UCB-MSCs did, suggesting that cell-to-cell interactions are not required [29]. Additionally, P-MSCs decreased NK cell immune responses [32]. It is known that, compared to bone marrow-derived mesenchymal stem cell (BM-MSCs), P-MSCs are more proliferative, resilient, and possess a higher potential for long-term growth [33, 34]. Compared to umbilical cord blood-derived mesenchymal stem cell (UCB-MSCs), P-MSCs are better at immunomodulation and create more colonies than UCB-MSCs separated from the same tissue because P-MSCs have a stronger potential for proliferation [29, 35].

In this study, owing to the distinguishing features of P-MSCs in regenerating ischemic tissue and regaining tissue function through immunomodulation and angiogenesis, the therapeutic benefit of P-MSCs was utilized for subjects with CLI. This is a report of a dose-escalation phase 1 study (IRCT ID: IRCT20210221050446N1) to evaluate the tolerability and identify the dose-limiting toxicities (DLTs) and maximum tolerated dose (MTD) of intramuscular P-MSC delivery in nine atherosclerotic and non-atherosclerotic patients with CLI.

## Materials and methods

### Patient recruitment and study design

Individuals with CLI who participated in the inpatient and outpatient vascular surgery services at Taleghani Hospital, whose primary treating vascular surgeons determined that they were not eligible for surgical or endovascular intervention but were considered suitable candidates, were offered to participate in this trial. Nine patients with CLI were included in the trial: two with diabetic foot and seven with TAO (Table 1).

**Table 1:** Demographic and baseline disease condition

Case	AGE	Diagnosis	Previous treatment for critical limb ischemia	Risk factors	Ischemic site/status
1	57	TAO	Minor amputation	ESH, BD	Right foot (II-4), left foot (III-5)
2	40	Diabetic foot	Minor amputation	ESH, DM	Right toe (II-4), left foot (III-5)
3	41	TAO	Sympathectomy	ESH, BD	Right foot (II-4)
4	55	TAO	Minor amputation	ESH, BD	Right foot (III-5)
5	61	Diabetic foot	Sympathectomy	DM, HC	Right foot (III-6)
6	57	TAO	Sympathectomy, major amputation	ESH, BD	Right foot (III-6)
7	66	TAO	Minor amputation, sympathectomy,	AS, BD	Right foot (III-6)
8	56	TAO	Minor amputation, sympathectomy	AS, BD	Right toe (III-5)
9	62	TAO	NO	AS, BD	Left foot (II-4)

HC: Hypercholesterolemia; ESH: ex smoke habit; BD: Buerger disease; AS: active smoker; DM: diabetus mellitus; TAO: thromboangiitis obliterans

The enrolled CLI patients had complete arterial occlusion without run-off of the lower extremities, as verified by conventional diagnostic angiography. Table 2 presents the inclusion and exclusion criteria for this study.

**Table 2:** Eligibility criteria for patients enrolled in the study

Inclusion criteria	Exclusion criteria
Written informed consent	Patients with CLI suitable for surgical or percutaneous revascularization
Male or female, age between 18 and 85 years	Patients with any acute/chronic inflammatory condition
Severe PAD (Rutherford class II-4, III-5, or III-6)	Major tissue loss in either leg or potential need for major amputation or revascularization within one month of study entry
Resting pain or ischemic ulcer/necrosis/gangrene	Severe heart failure, respiratory failure, liver failure, kidney failure, or poor general condition so that the patient cannot tolerate stem cell transplantation
None surgical nor endovascular option	Diabetes mellitus with HbA1c > 7%
	Active malignancy or history of malignancy within five years prior to study entry
	Evidence of active infection
	Stroke or acute myocardial infarction
	Chronic kidney disease (Stage 4 and 5)
	Any condition that leads to the patient not being able to express pain and complain about the disease (such as mental retardation, alcohol consumption)

Based on the trial entrance order, eligible patients were randomized into one of the two P-MSC dosing cohorts. A 3 + 3 dose escalation approach was adopted, with three–six patients in each P-MSC dose cohort: 20 × 10^6^ cells (low dose) and 60 × 10^6^ cells (high dose). Dose levels were chosen using data from prior clinical studies. Three participants were assigned to the first group. When the individuals conducted their day 14 evaluations, an unbiased safety monitoring board reviewed the safety evidence to determine whether an additional three subjects could be recruited in the second group until the MTD was achieved. If no more than one patient had DLT within the first 14 days of follow-up, the number of subjects in the second group was increased to six.

A DLT was described in this study as a grade 2 toxicity or any grade 3 toxicity expected to be attributable to P-MSC administration that did not resolve during the 14 days. The highest P-MSC dosage for which the prevalence of DLTs was less than or equal to one case was regarded as the MTD. If two or more subjects developed a DLT during the 14 days of administration, the MTD was considered to have been exceeded.

### Preparation of P-MSCs

P-MSCs were harvested from placental tissue acquired from a full-term delivery with written informed consent from a healthy mother and delivered fresh tissue. After successful vaginal birth at a maternity hospital, the placenta was obtained from informed, healthy women (Additional file 1: Table S3). Following the initial passage and on the final cultivated MSCs, the cells were assessed for sterility, mycoplasma presence, and endotoxin concentration. Screening of placental sources for viral infections was also performed. The cell viability was determined using the trypan blue exclusion method.

According to the current GMP (Good Manufacturing Practice) standards, all steps were carried out in a GMP-grade clean room facility. The fresh placenta was rinsed with PBS, divided into small pieces, and washed with 0.9% sodium chloride solution to remove any remaining blood before incubation for three hours at 37 °C with GMP-grade collagenase NB6 at 1 mg/mL (SERVA Electrophoresis GmbH). Following the addition of 0.9% sodium chloride solution, the mixture was shaken and centrifuged.

The supernatant was withdrawn and the cell pellet was cultured on MSC complete medium with 10% pharmaceutical grade Australian origin fetal bovine serum (ATOCEL). Primary cultures were kept in a 37 °C humidified 5% CO_2_ incubator for one week in small digested fragments. Non-adherent cells were eliminated by transferring the culture medium twice a week. Adherent MSCs were passaged using the animal origin-free TrypLE express enzyme at approximately 80% confluence to obtain sufficient quantity of MSCs for further use. The P-MSCs were suspended in 100 ml of normal saline that had been enriched with human serum albumin for each administration.

According to the International Society of Cell Therapy (ISCT), confirmatory tests were carried out, including flow cytometry (CD34, CD45, CD29, CD90, and CD105) (Additional file 1: Fig. S1) and MSC multilineage differentiation (into bone, cartilage, and fat).

### Implant procedure

P-MSCs were administered under spinal anesthesia (3 patients) or IV sedation (6 patients) to relieve pain. P-MSC treatment was administered at two time points, eight weeks apart. The total amount of the suspension was injected intramuscularly using a 30 G needle to a depth of 1–1.5 cm and an area of 10 × 6 cm (30–40 sites with 0.5–1.0 ml of P-MSCs per site) on each occasion. The injections were distributed as follows: the knee to the ankle was divided into five equal parts in four lines (the anterior, anterolateral, and superficial posterior compartments of the leg), and six points were identified at the dorsum of the foot, and the surroundings of the wound (if there was a wound).

Patients received 100 mg hydrocortisone before injection to avoid side effects such as allergies. Oxygen saturation was measured 30 min before and 6 h after the injection of P-MSCs throughout the dosage.

### Outcome measurement and follow-up

To assess the safety and effectiveness of P-MSC injections in the enrolled participants, we assessed medical records, electrocardiogram (ECG) results, vital signs (respiration, heart rate, blood pressure, oxygen saturation, and body temperature), blood chemistry, physical examination reports, digital photographs of wounds, and angiography at baseline and at the months follow-up.

The primary safety evaluations that followed repeated doses of perinatal tissue MSC transplantation in no-option CLI patients, including tracking and documenting all possible adverse events (AEs) according to the National Cancer Institute Common Terminology Criteria for Adverse Events (NCI CTCAE; Version 5.0), were the main outcomes of the study.

In addition to the usual safety laboratory measures, the levels of a few pro-inflammatory cytokines such as interleukin-1 (IL-1), tumor necrosis factor-α (TNF-α), and interferon-γ (IFN-γ) were assessed to determine the immunological response to P-MSC transplantation (ELISA R&D, USA). Flow cytometry was used to compare the lymphocyte profiles before and after P-MSC administration in terms of CD4, CD8, and CD25 levels.

In this study, in addition to investigating the safety of placental mesenchymal stem cell administration, efficacy endpoints were also investigated, including an increase in the ankle-brachial pressure index (ABI), maximal walking distance, vasculogenesis, relief of rest pain, healing of ulceration, and prevention of major amputation in the target limb.

**Fig. 1:**
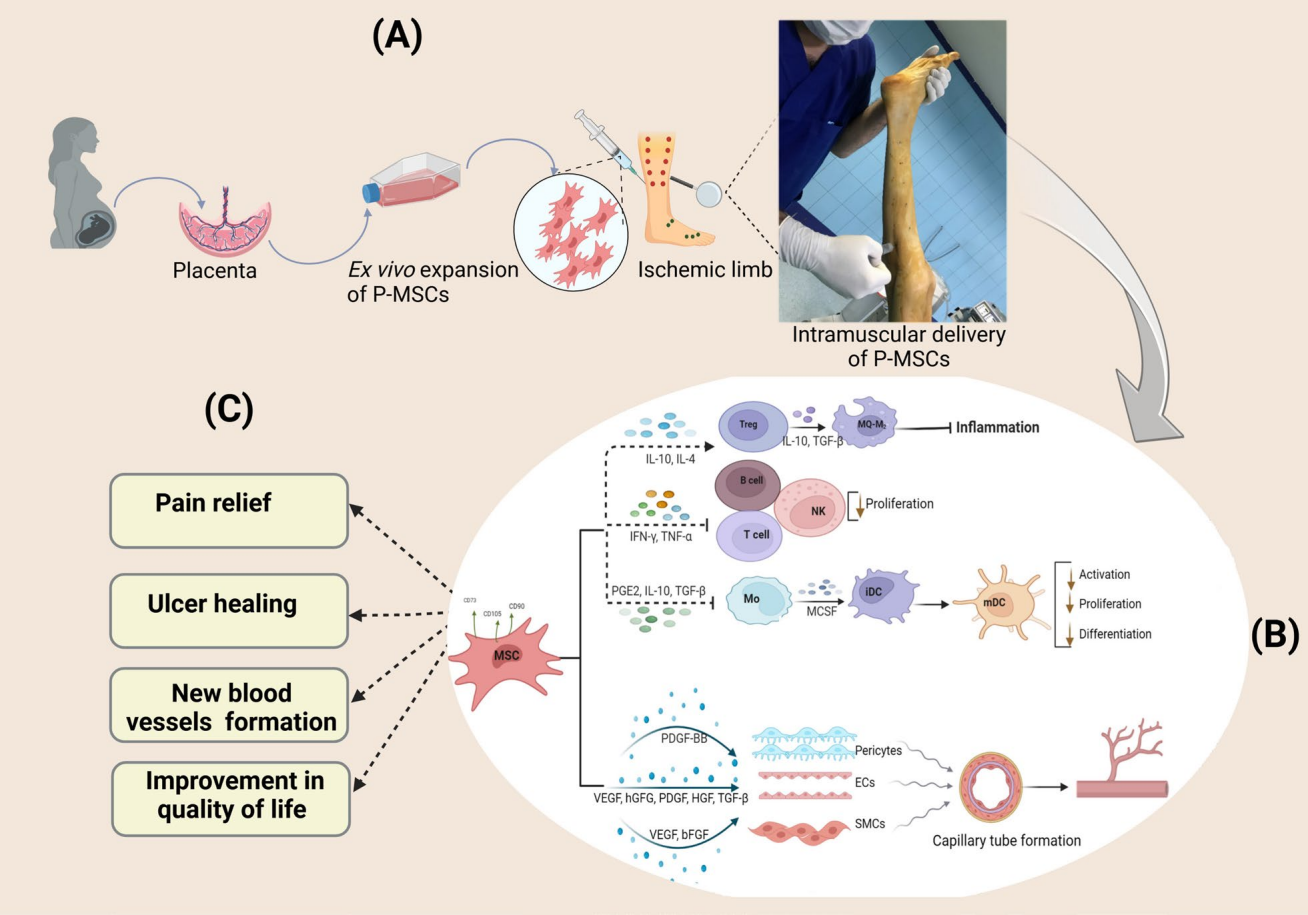
Clinical trial methodology for intramuscular administration of P-MSC. **A** P-MSC injected to the ischemic limb following isolation and in vitro expansion, **B** P**-**MSCs suppress inflammation and promote immunomodulation by secreting immunomodulatory cytokines, which stimulate the induction of M2 macrophages and increase the number of circulating regulatory T cells, resulting in an increase in interleukin IL-10 and resolution of inflammation. Additionally, MSCs release factors that promote angiogenesis directly. **C** Efficacy outcomes of P-MSC administration in patient with CLI

The visual analog scale (VAS) was used to evaluate pain, ranging from 0 for the best (totally resolved) to 10 for the most painful condition. Wound healing was evaluated using digital photographs of the ulcers. Objective criteria for limb ischemia included ABI measurements (*Dopplex*® Ability Automatic ABI System, *HUNTLEIGH*, USA) and quality of life (vascular quality of life questionnaire-6). The number of apparent collateral vessels was also monitored using conventional angiography before and six months after P-MSC administration. Figure 1 represents summary of P-MSCs clinical trial procedure.

### Statistical analysis

Individual data are described using frequency graphs and tables. Categorical data were summarized as counts and percentages. All data from participants who withdrew prematurely from the study were included in any analysis where possible. The data were transferred to a statistical package for analysis (GraphPad Prism 9.0; GraphPad Software, La Jolla, CA, USA). The Wilcoxon nonparametric paired test was used to compare inflammatory cytokine levels, lymphocyte profiles, blood chemistry, and collateral grades on conventional angiography between the baseline and 6 months after the intervention. One-way ANOVA or Friedman test was applied to compare the variables (VAS rating scores, ABI, maximal walking distance, and quality of life) between different times before and after the intervention. Differences were considered statistically significant at *p* < 0.05. As the data were inadequate for statistical analysis, the rates of amputation and wound healing were described descriptively.

## Results

### Characteristic of patients

**Fig. 2:**
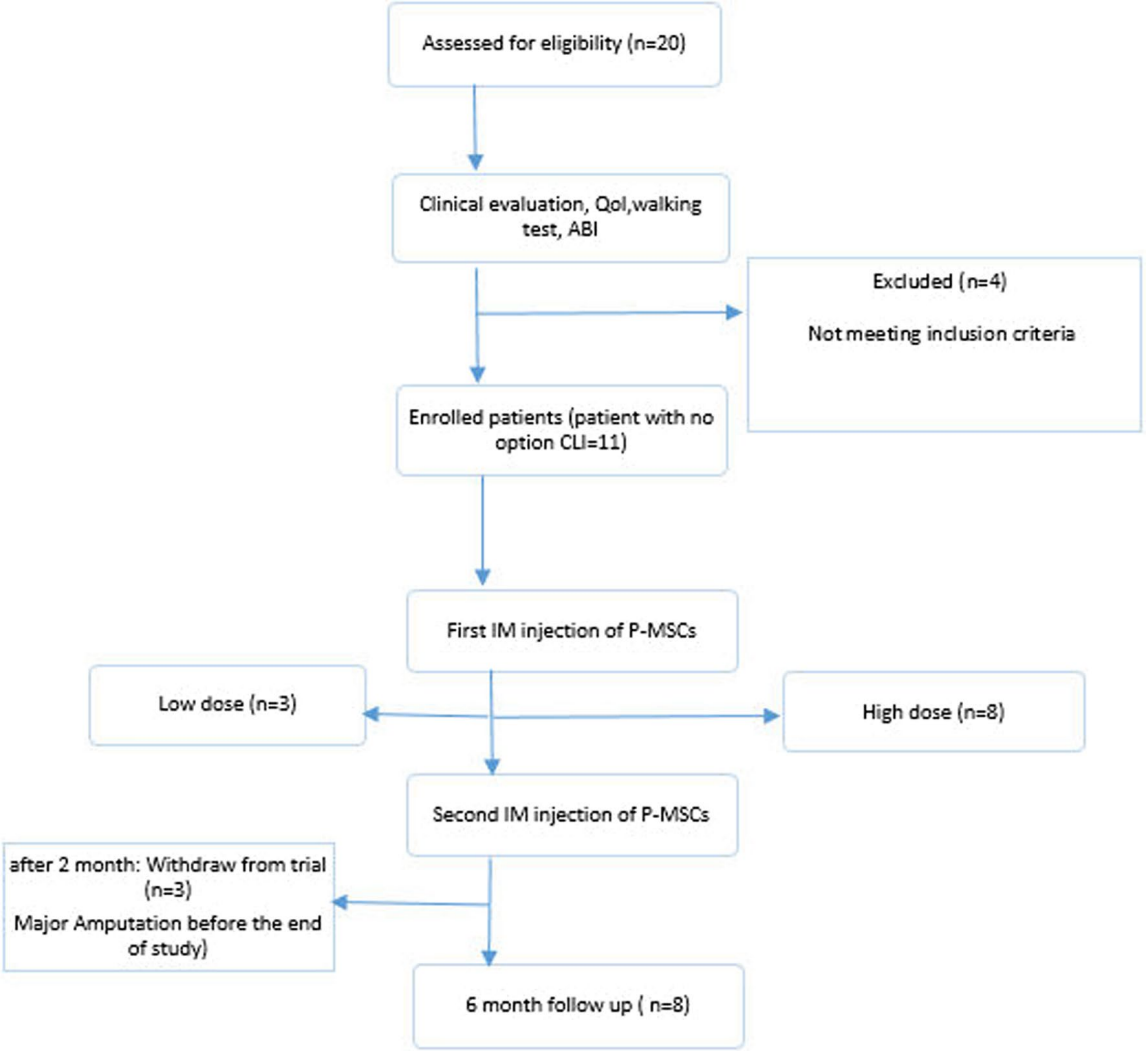
Flowchart: 15 patients recruited in the study and allocated to two groups (low-dose group = 3; high-dose group = 8)

From April 2022 to August 2022, 15 patients with CLI were recruited for the clinical trial to determine their eligibility for study enrolment. Of these, 11 patients received treatment with P-MSCs, and eight patients completed the trial. Three patients were excluded from the study because of clinical worsening (Fig. 2). The subjects were followed for six months after receiving the cell injections. All the patients had CLI, and the underlying cause was either TAO or ASO. The average age of the participants was 55 years and all were males. All patients with TAO were smokers. More than 90% of the ischemic limbs (10/11) had necrotic feet or non-healing ulcers (Rutherford classes II-3, III-5, and III-6), and 54.5% (6/11) had already undergone minor or major amputation. Table 3 shows a comparison of the clinical outcomes at baseline and 6 months after P-MSC implantation.

**Table 3:** Comparison of clinical outcome on baseline and 6 months after implantation of P-MSCs

Case	Cell dose	Walking distance		Rest pain		ABI		Vascular quality of life (questionnaire-6)		Ischemic site status	
		Day 0	Day 180	Day 0	Day 180	Day 0	Day 180	Day 0	Day 180	Day 0	Day 180
1	Low	400	2000	8	0	Right: 1.57	1.56	12	24	Toe gangrene (amputated) surgery	Well improved
						Left: 1.09	1.09				
2	Low	200	700	7	1	Right: 0.8	1.22	11	20	Toe necrosis/history of minor amputation	Minor amputation, well improved
						Left: 0.7	1.21				
3	Low	N/A	N/A	9	0	1.13	1.43	10	19	Non-healing ulcers	Ulcer healing, well improved
4	High	300	1500	8	1	1.25	1.16	12	22	Extensive gangrene with impending amputation/history of minor amputation	Minor amputation (day 30), well improved
5	High	N/A	N/A	9	N/A	0.78	N/A	9	N/A	Extensive gangrene with impending amputation	Major amputation (day 70)
6	High	10	10	9	2	0.87	0.92	9	20	Extensive gangrene with impending amputation	Minor amputation (day 90), well improved
7	High	0	100	8	2	1.16	0.8	11	19	Extensive gangrene with impending amputation	Minor amputation (day 40), well improved
8	High	200	1000	7	1	1.17	1.3	13	20	Non-healing ulcer	No change
9	High	250	1000	8	2	0.98	1.21	15	23	Non-healing ulcers at dorsum of the foot/gangrenous toes	Ulcer healing, No change

### Safety evaluation of P-MSC therapy

The MTD was not reached after P-MSC treatment, as no cases of DLT or AEs leading to discontinuation of P-MSC treatment were reported in this trial. Intramuscular injection of all doses of P-MSCs, including the maximum tested dose of 60 × 10^6^ cells, was well-tolerated, with no notable differences observed between the two groups. After the administration of P-MSCs, no patient experienced infection, hemorrhage, or other issues associated with the microbiological state of the cells or procedure-associated difficulties.

Two patients experienced moderate post-injection diarrhea a day after cell therapy. In addition, two patients developed minor pruritus. The diarrhea improved on its own, and pruritus disappeared after one day of antihistamine therapy. There was one adverse event due to disease progression that was unrelated to the cell treatment; one patient received below-knee amputation of the treated leg three months after cell administration. During the follow-up phase, the physical evaluation and vital signs did not change from baseline.

All patients underwent routine hematological and biochemical testing, both before and after cell treatment. The results demonstrated that the test improved over time. Among which FBs (fast blood sugar) showed a significant decrease (*P* ˂ 0.0273) (Additional file 1: Table S2).

**Fig. 3:**
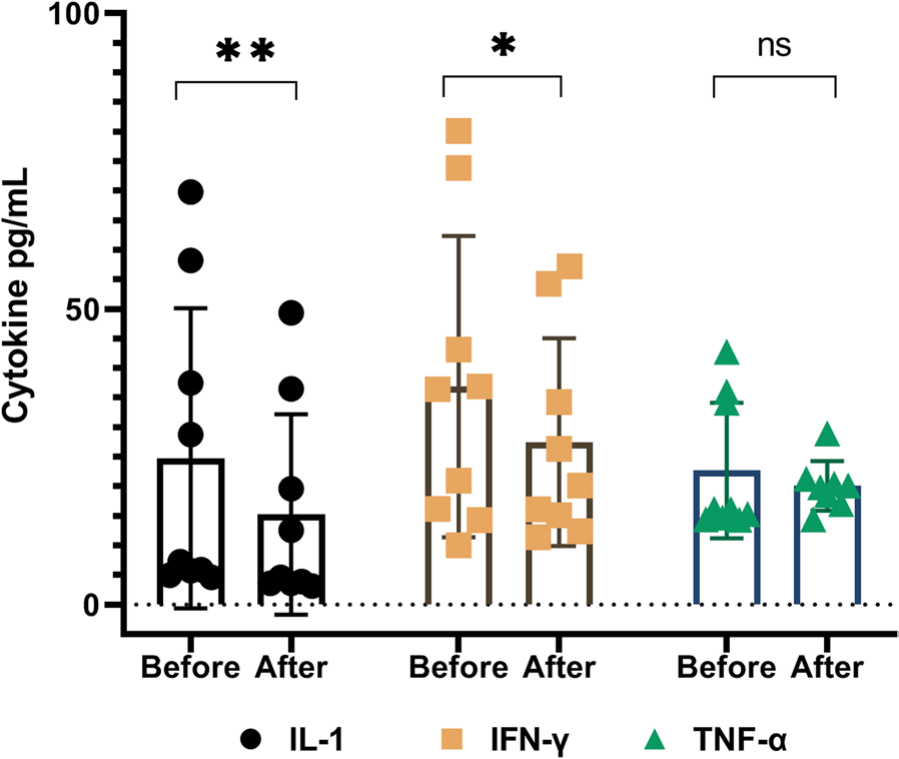
Change in patients’ serum inflammatory cytokines levels on days 0 (baseline) and 6 months after the first infusion. Analysis of biomarkers on before (baseline) and 6 months after the first infusion demonstrated a significant reduction in IL-1, and interferon-γ (IFN-γ) in all patients with CLI. Serum levels of tumor necrosis factor alpha (TNF-α) decreased in four cases, but the differences were not statistically significant. *P < 0.05; ns, not significant

The immunological profiles (IL-1, IFN-γ, and TNF-α) (CD4, CD8, and CD25) of patients with CLI were evaluated before and 6 months after P-MSC administration in this study. The results demonstrated a statistically significant reduction in IL-1 and IFN-γ serum levels over a 6-month follow-up period after P-MSC therapy (*P* ˂ 0.05). However, there was no statistically significant reduction in serum TNF-α concentrations (*P* > 0.05) (Fig. 3).

**Fig. 4:**
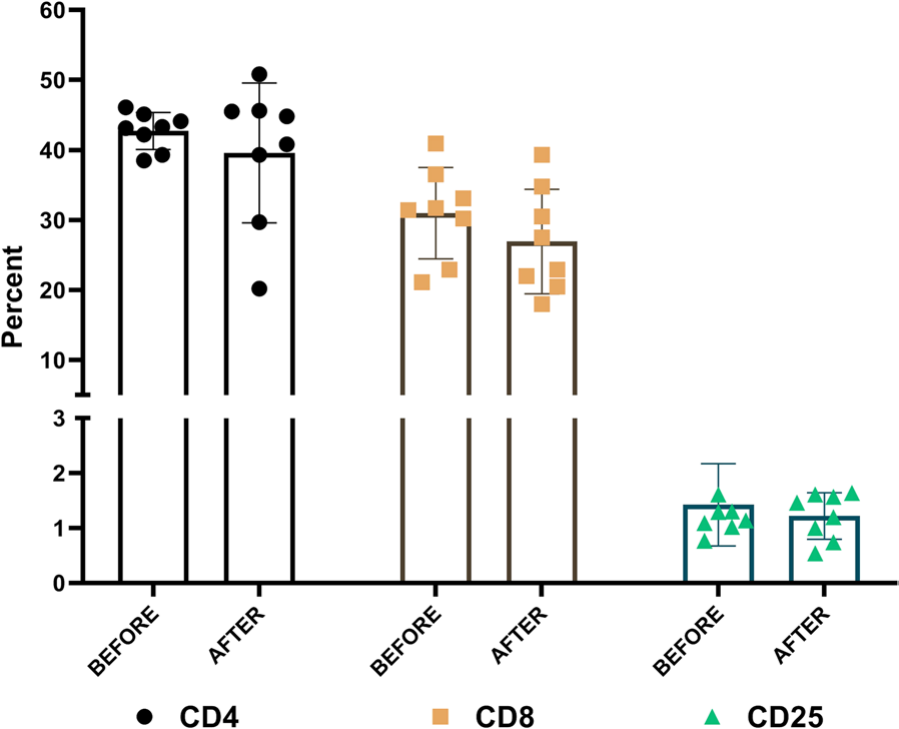
Measured markers of CD4 and CD8 and CD25 on patients with CLI at screening and 6-month follow-up after P-MSC implantation

Patient samples were analyzed using flow cytometry for CD4, CD8, and CD25 markers before and after treatment with P-MSCs (Fig. 4) (Additional file 1: Table S1). According to the data obtained for the various T lymphocyte subsets, there was no noticeable variation in the blood lymphocyte profile of CLI participants, implying that the injected allogeneic cells did not result in T cell proliferation in vivo (Additional file 1: Fig. S1). Altogether, these findings show that the immune profile of CLI patients was not adversely influenced by allogeneic P-MSC administration and was completely safe.

### Secondary outcome measures of P-MSC therapy

#### Angiogenesis

**Fig. 5:**
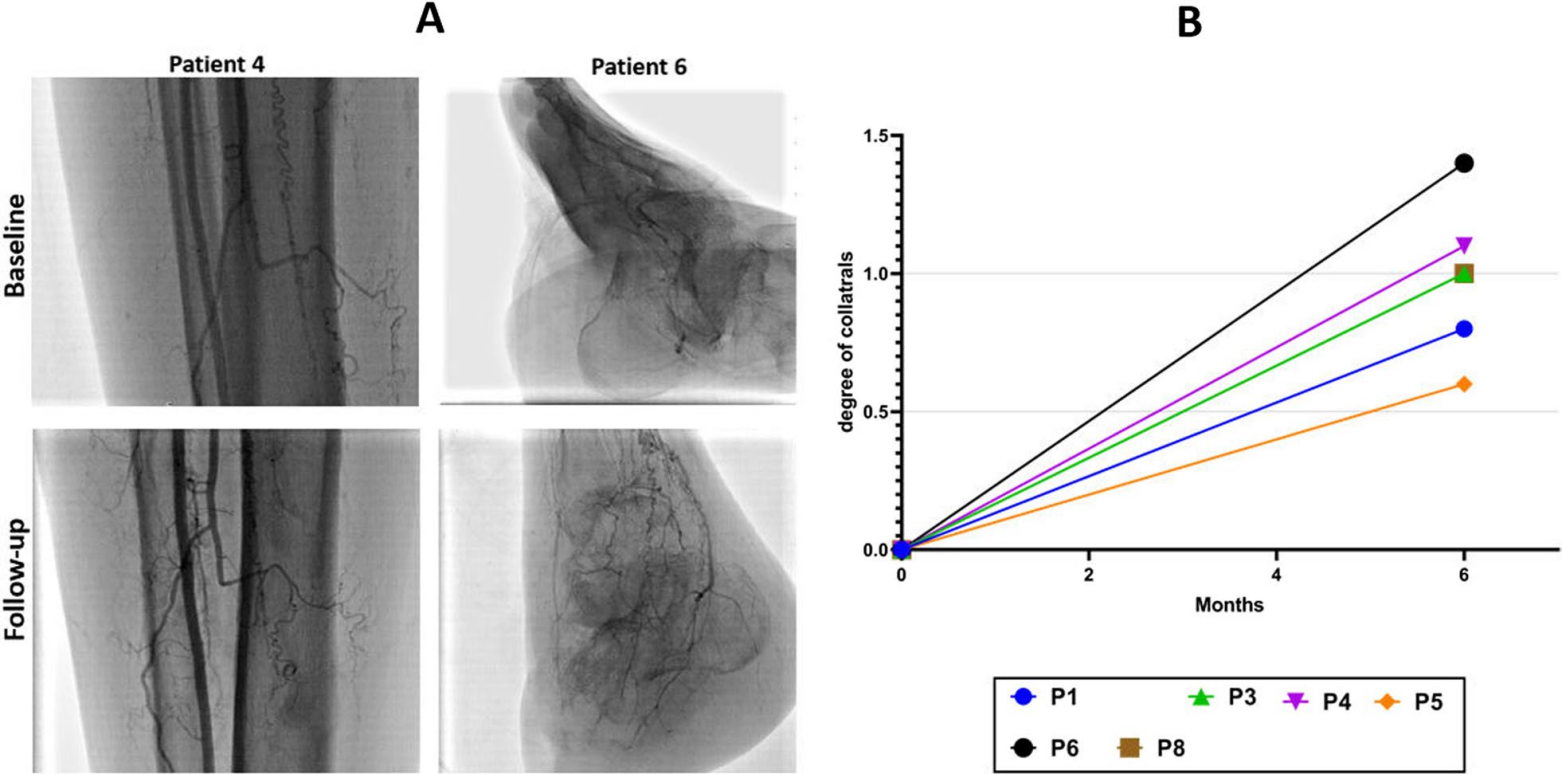
Changes in collateral artery development on angiography 6 months following implantation of placenta-derived mesenchymal stem cells (P-MSC). **A** Collateral vessel formation was increased at 6 months after P-MSC administration. **B** The degree of collateral vessel formation was ranked as 0, + 1, + 2, and + 3 according to the number of collaterals (*P* value = 0.0625)

We evaluated arterial angiography data 6 months after cell injection and classified the degree of collateral vessel development as + 0 (no collateral circulation), + 1 (slight collateral circulation), + 2 (moderate collateral circulation), and + 3 (high collateral circulation) [36, 37]. Angiographic evaluation was performed by three cardiologists who were blinded to the patient's clinical condition. After cell infusion, collateral vessel formation scores were elevated to 0.98 + 0.3 (Fig. 5). However, angiography was unable to demonstrate these new small vessels in all but a few cases.

#### Pain relief and physical functioning

**Fig. 6:**
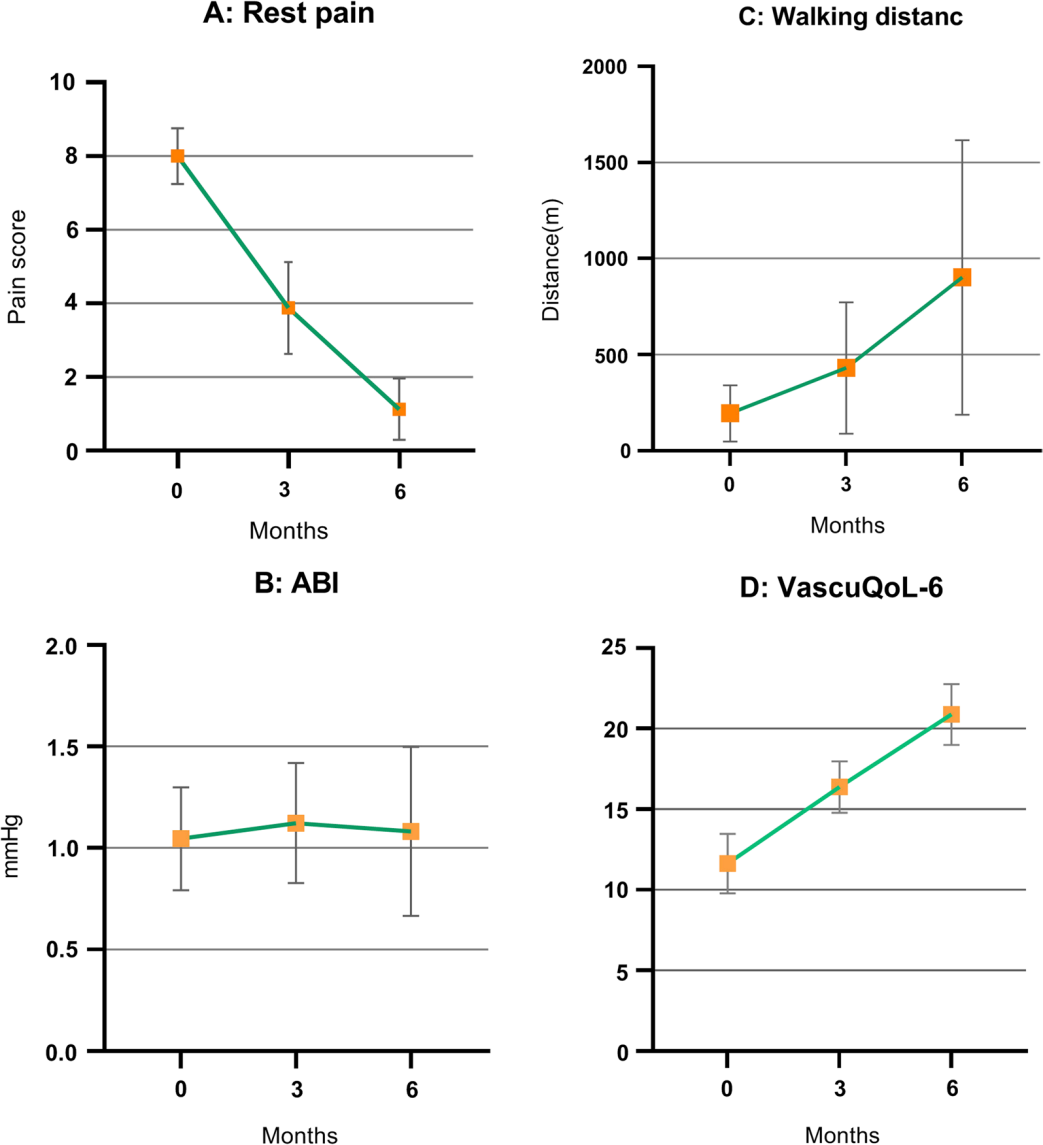
Efficacy parameters: **A** Ischemic rest pain score measured by verbal analogue scale: 0 = no pain, 10 = worst pain in patient’s life, *P* < 0.0001. **B** Ankle brachial index of the treated limb, *P* = 0.1228. **C** Walking distance, *P* = 0.0017. **D** Vascular quality of life questionnaire-6, *P* < 0.0001

At the beginning of the trial and at the 1-, 3-, and 6-month follow-up visits, the rest pain was assessed using VAS (Table 3). Among the limbs that completed the follow-up period, all patients showed significant pain improvement. The mean VAS baseline score was 8 (± 0.75), which improved to 3.87 (± 1.24) after 3 months and 1.125 (0.83) (*P* ˂ 0.0001) after 6 months (Fig. 6A).

As shown in Fig. 6B, the mean ABI in the all treated limbs increased steadily following cell infusion from 1.04 (± 0.25) mmHg at baseline to 1.12 (± 0.29) mmHg at 3 months and then decreased to 1.08 (± 0.41) mmHg at 6 months in all the treated limbs (Table 3). The improvement in ABI seen as early as 3 months after cell therapy did not persist throughout the full follow-up period.

In six patients, the treadmill test performed to determine the maximum walking distance improved significantly (*P* ˂ 0.001) (Table 3) (Fig. 6C). The remaining patients had amputated toes, feet, or painful ulcer lesions that prevented participation in the treadmill test.

The VascuQoL-6 survey was used to subjectively assess changes in leg symptoms and benefits after cell infusion (Fig. 6D). There was a statistically significant (*P* ˂ 0.0001) improvement in health-related quality of life in the post-treatment period. It is worth mentioning that the QOL scores in the group of patients with a recent safety visit were similar to those measured at the latest follow-up visit (6 months). Our findings demonstrated that following cell implantation, physical function and rest pain improved significantly in all participants. One patient demonstrated significant improvements in the injected leg despite major amputation before six months of follow-up. All participants were satisfied with their outcomes, and it was observed that relief from rest pain resulted in a considerable increase in performance in activities, especially continuous walking, and a significant decrease in analgesic utilization.

#### Ulcer healing and Limb salvage

**Fig. 7:**
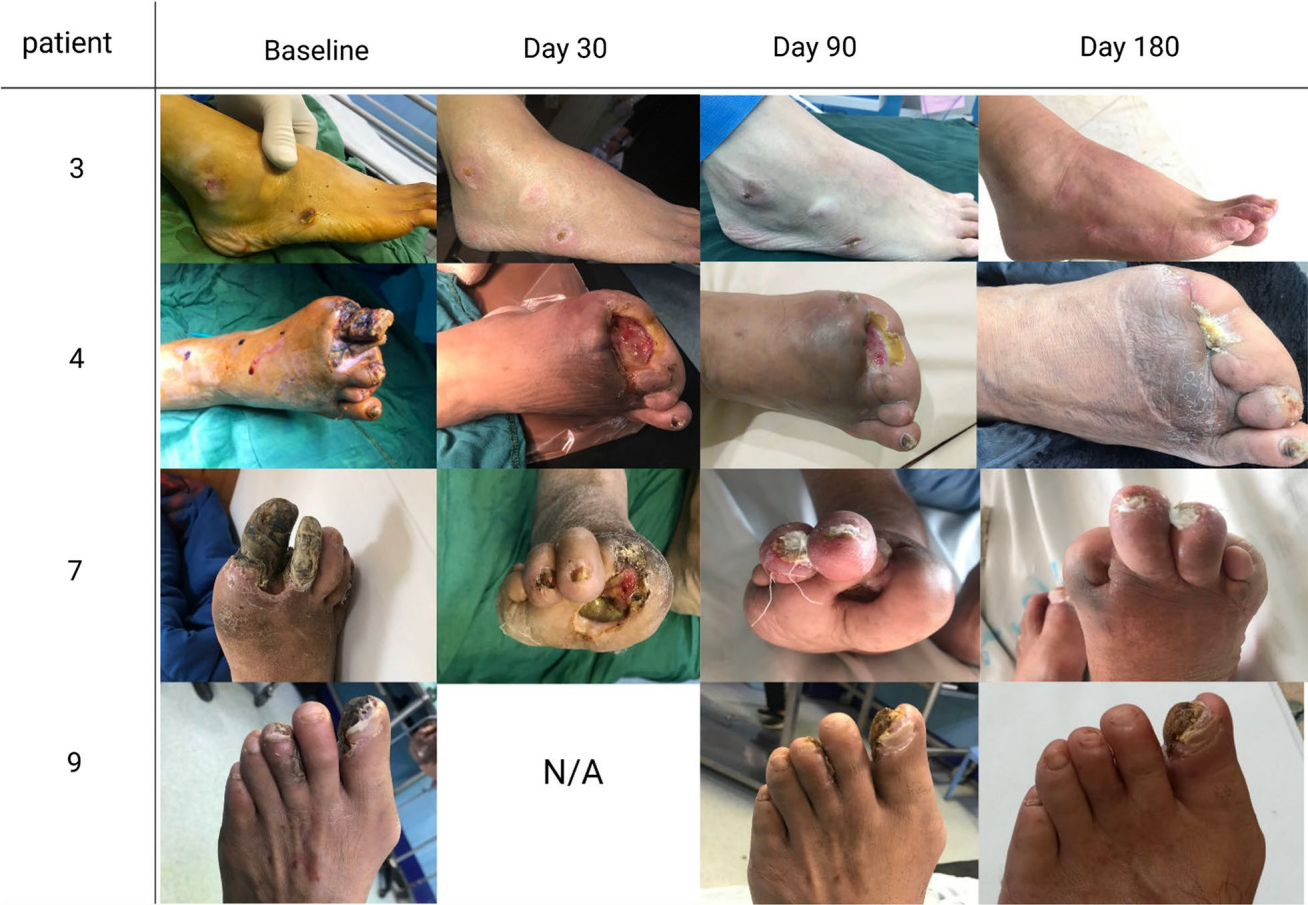
Representation of the treated limbs showing the progression of ischemic ulcer/gangrene throughout 6-month follow-up. Patient 3: a 41-year-old man with thromboangiitis obliterans (TAO), Rutherford IV presented with non-healing ulcers on the ankle and lateral midfoot healed completely at 6 months after P-MSC implantation. Patient 4: a 55-year-old man with TAO presented with gangrene of the first and second toes, and a history of amputation of the 3th and 4th toes showed clear healing of ischemic necrosis on foot after amputation at 6 months after P-MSC implantation. Patient 7: A 66-year-old men, TAO with Rutherford-III, initially advanced gangrene and wound infection who diagnosed as needing major amputation, get minor amputation after cellular therapy and whole ulcer surface measurement was largely decreased at 6 months after P-MSC implantation. Patient 9: a 62-year-old man with Rutherford IV (TAO) presented with non-healing ulcers at dorsum of the foot and gangrene of the toe, showed clear healing of ulcer. Gangrene did not progress during the 6-month follow-up

Ten extremities completed the 6-month follow-up study. At baseline, nine limbs had non-healing sores, necrosis, and gangrene. Throughout the 6-month follow-up period, one limb demonstrated significant healing of the ulcer, and two limbs with ulcers and gangrenes did not progress and remained entirely dry. We performed minor amputations in one patient before cell treatment and five patients after P-MSC implantation, whose wounds healed after amputation, and the total ulcer surface measurement was largely reduced (Fig. 7).

At the six-month follow-up, the amputation rate in this trial was 40% (4 of 10 limbs). Of the nine patients, four underwent amputation, three of whom were diagnosed as needing major amputation. Referring clinicians concur that the first therapeutic approach is no longer effective following P-MSC therapy. Hence, they underwent minor amputation. Only one patient underwent major amputation (due to superinfection at the gangrene site) on day 70 after cell therapy and was excluded from the trial before the end of follow-up.

## Discussion

In ischemic lesions in CLI patients, normal vascular regeneration mechanisms, including angiogenesis and arteriogenesis, do not take place [13]. To enhance blood circulation and the quality of care for life-threatening illnesses, it is critical to consider alternative treatment options [9, 38]. Preclinical research has shown that P-MSCs can infiltrate damaged tissues, release anti-inflammatory factors, and promote angiogenesis [26, 27, 30]. Several phase I through III clinical trials using MSCs administered by intramuscular injection for critical limb ischemia are now underway, with encouraging outcomes [20]. Nevertheless, only three phase I clinical studies that examined the tolerability and viability of P-MSCs for CLI have published the final results from these trials [20, 39–41]. Wu et al. conducted a dose-escalation phase I clinical investigation on the safety of P-MSCs in PAD patients. Following P-MSC delivery, there were initial signs of wound regeneration, improved peripheral blood flow, and decreased levels of biomarkers of vascular damage. They concluded that P-MSCs were generally safe and beneficial for patients with PAD [41].

No clinical research has documented the safety and effectiveness of utilizing P-MSCs to treat patients with non-atherosclerotic PAD. Therefore, in this primary clinical trial, we assessed the safety, feasibility, and tolerability of prenatal MSCs as potential treatments for patients with CLI. Our findings demonstrated that, following cell injection, physical function and clinical complications improved significantly in all participants. Most of the recovered patients had a dramatic response—2 to 3 month after the first MSC injection that persisted during the 6-month follow-up assessment. Despite the small patient population, the efficacy outcomes for some patients with advanced disease stages are highly encouraging.

In this dose-escalation phase I experiment, two cell doses (20 million and 60 million MSCs) were tested until the MTD was established, as the optimal number of cells required for angiogenesis remains unknown. Few studies on cell doses have been conducted, and such studies will be critical for optimizing the administration of enlarged populations of stem cells or marker-selected stem cell populations MTD was established. MTD was not achieved following P-MSC therapy because no occurrences of DLT or AEs leading to P-MSC treatment discontinuation were recorded in this experiment. All dosages of P-MSCs, including the highest tested dose of 60 × 10^6^ cells, were well tolerated intramuscularly with no significant differences between the two groups. In contrast, the immunological profiles of patients with CLI did not significantly differ between baseline and the 6-month follow-up, suggesting that the delivery of allogeneic P-MSCs was completely safe. This finding is consistent with prior safety findings from comparable clinical studies [42–47].

We selected appropriate patient populations for the transplantation of stem cells in this clinical study, as there is a great deal of diversity in the capacity of patients to establish significant vascular repairs [18]. Simultaneous patient disorders or atherogenic risk factors, on the other hand, influence either stem cell abundance or function [48]. To show such variation between patients, we also included patients with non-atherosclerotic PAD along with participants with ASO. Accordingly, the clinical outcomes improved more dramatically in patients with TAO. Moreover, it is important to assess the severity of ischemia in individuals selected for cellular therapies. Walter et al. discovered that patients with Rutherford stage 6 failed to respond to therapy, whereas those with stages 4–5 did respond [49]. Similarly, the majority of patients in this study belonged to Rutherford stages 4–5, showing the highest level of recovery. Despite remarkable improvement in one ASO patient's injected with Rutherford stage 6, he underwent major amputation before the conclusion of the follow-up period.

We utilized IM injection in this trial because it effectively conveys a large number of cells to ischemic sites, as reported in preexisting cell-based treatments for CLI [20]. The advantages of IM delivery include a more practicable and less invasive approach [17], creation of a cell depot with paracrine action in the ischemia region, and ease of use [50]. A clinical study that compared intra-arterial and intra-arterial plus intramuscular cell delivery reported that intramuscular cell treatment dramatically improved ulcer healing [51, 52]. In this clinical trial, the levels of major inflammatory cytokines (IL-1, IFN-γ, and TNF-α) were assessed to determine the safety of the P-MSC injection. Levels of IL-1 and IFN-γ were significantly lower after MSC administration. Although it has been previously shown that MSCs can regulate the inflammatory response in patients with CLI [37, 40, 44, 53], the precise mechanism by which MSCs exercise their therapeutic benefits is not yet entirely understood. MSCs inhibit pro-inflammatory cytokine release from infiltrating immune cells [54]. Additionally, MSCs can inhibit regulated cell death by secreting hepatic growth factor (HGF), keratinocyte growth factor (KGF), and angiopoietin-1, and decreasing TNF-α levels [55–57].

Although this trial was not powered for effectiveness criteria measures, we found clinically substantial improvements in limb blood circulation and severity of limb ischemia. This was demonstrated by a substantial improvement in rest pain, blood flow, and maximum walking distance after P-MSC implantation. Pain scores showed a decreasing trend after treatment with P-MSCs in both dose groups, up to a decrease of 4.5 units. ABI, which is considered a marker of tissue perfusion, increased over the first trimester. However, this increasing trend was not significant in the second trimester after the injection (mean baseline ABI: 1.04; 3-month mean ABI: 1.12; 6-month mean ABI: 1.08.4, *P* = 0.12).

In this trial, all patients showed improved symptoms on the QoL questionnaire and reduced analgesic usage. Interestingly, one patient experienced dramatic improvement in the injected leg despite major amputation before the end of the follow-up period. It is worth emphasizing that all patients acknowledged and were informed of the significant clinical benefits of the treated limb.

In this clinical trial, angiographic evaluation was performed before and after cell infusion and revealed that collateral vessel formation scores were elevated. However, these changes were not significant (*P* value = 0.062). The improvement in microcirculation could be due to vessel collateralization, as demonstrated in some patients using angiography. The P-MCS treatment has the potential to accelerate this natural process. Various ideas have been proposed regarding the creation of new vessels under ischemic conditions. MSCs thrive in hypoxic and ischemic environments according to the first hypothesis [10, 58]. The second theory insists on the paracrine effects of MSC via proangiogenic chemokines and cytokines; to rephrase it, they act as activators of endothelial progenitor cells that promote the development of new vessels or collateralization [59, 60].

One of the main outcomes of effectiveness in this study was the prevention of major limb amputation. We were able to avoid or perform minor amputations rather than major amputations in the patients with CLI. In the study by Gupta et al., the number of ulcer healings and amputations was comparable in both groups (placebo and BM-MSC). It is possible that allogeneic BM-MSC treatment did not provide significant benefit to severely ill patients who were facing amputation owing to the advanced form of their condition [44]. In another trial, Walter et al. [49] required all four participants with severe gangrene and probable amputation (Rutherford class 6) to undergo amputation above the ankle within the first three months of the study. In contrast, in our trial, among the four patients with impending amputation, major amputation did not occur in two TAO patients who underwent minor amputation, and one patient with ASO seemed to have an extended amputation-free interval. The one reported below-the-knee amputation (BKA) within the initial 3-month period was in a 61-year-old man with ASO, who had recently undergone major amputation of the opposite leg related to disease progression.

In addition, considerable improvement or complete healing was observed in all patients with ischemic ulcers. Even when we had to perform minor amputations in a few patients before or after P-MSC implantation, the wounds healed after amputation, and the measurement of the total wound area was greatly reduced.

Our study had a number of caveats and limitations. This study was not powerful enough to determine whether high doses of P-MSCs were more effective than low doses of P-MSCs.

ABI improvement was the primary outcome of several stem cell studies on CLI; however, there was no noticeable difference between baseline and follow-up. Yet, other secondary endpoints, such as amputation-free interval extension, wound healing, and rest pain, showed substantial improvements [49, 60, 61]. These clinical trials revealed that ABI level is not a reliable indicator of the long-term efficacy of stem cell angiogenic treatment, and differences in the severity of CLI may explain some of the observed variations in perfusion changes between these investigations [44].

In this trial, the collateral vessel formation scores changes were not significant. Part of the reason is the small number of patients in this study. The other part could be the low resolution of the angiography method used in this clinical trial. It should be mentioned that there is a need for more accurate methods to check the collateralization, such as magnetic resonance angiography (MRA) and digital subtraction angiography (DSA), which can show the formation of new vessels with higher accuracy [36, 37]*.*

Because the number of injected limbs was insufficient to conduct a statistical analysis, data on the amputation-free survival rate and ulcer healing were reported descriptively.

Despite the immunomodulatory and anti-inflammatory characteristics of MSCs, there are emerging concerns regarding their tumorigenic potential owing to their innate predisposition to migrate to injured tissues and inflammatory regions [62]. In this regard, the pre-existing microenvironment may influence MSC behavior, causing them to acquire supportive properties for cancerous cells. In previous clinical studies, no malignancy was observed or persisted because of therapeutically delivered MSCs. Nonetheless, there may be risks associated with the establishment and proliferation of undiscovered or lingering cancer cells in the body. To better understand tumorigenesis in patients who receive allogeneic P-MSCs, prolonged follow-up is required [63].

In this study, we followed the patients for 6 months in terms of clinical and preclinical outcomes, which seems to be short, since some criteria such as the results of angiogenesis, ABI, relief of rest pain, healing of ulceration, and prevention of major amputation in the target limb may change in the long term. In other words, most of the results will be more accurate during a more thorough follow-up.

## Conclusions

In conclusion, our observations from this early clinical trial indicate that intramuscular treatment with high doses of P-MSCs is safe and well tolerated. Despite our encouraging results, we cannot claim that MSC treatment is beneficial and safe for patients with CLI. To fill this knowledge gap regarding the therapeutic properties of MSCs for critical limb ischemia, additional large randomized clinical trials are necessary.

## Supplementary Information

**Additional file 1: Figure S1** Flow cytometric characterization of PM-MSCs. The cells were negative for CD45, CD34. They displayed positive expression for MSCs markers; CD105, CD90, and CD29. **Figure S1.a** Placenta 1. **Figure S1.b** Placenta 2. **Figure S2** Flow cytometry of CD4 and CD8 and CD25 on patients with CLI. **a** Patient 2, A. CD4/CD25 before P-MSC injection, B. CD4/CD25 after P-MSC injection, C. CD4/CD8 before P-MSC injection, D. CD4/CD8 after P-MSC injection. b. Patient 4, A. CD4/CD25 before P-MSC injection, B. CD4/CD25 after P-MSC injection, C. CD4/CD8 before P-MSC injection, D. CD4/CD8 after P-MSC injection. c. Patient 9, A. CD4/CD25 before P-MSC injection, B. CD4/CD25 after P-MSC injection, C. CD4/CD8 before P-MSC injection, D. CD4/CD8 after P-MSC injection. **Table S1** Comparison of CD4, CD8, and CD25 markers results on baseline and 6 months after implantation of P-MSCs. **Table S2** Laboratory findings before the first and after the last cell infusions. **Table S3** Characteristics of placenta donors.

## Data Availability

All data generated or analyzed during this study are included in this published article.

## Abbreviations

ABI: Ankle-brachial index
AEs: Adverse events
ASO: Atherosclerosis obliterans
BM-MSCs: Bone marrow-derived mesenchymal stem cells
BKA: Below-the-knee amputation
CLI: Critical limb ischemia
Cr: Creatinine
DFUs: Diabetic foot ulcer
DSA: Digital subtraction angiography
ECG: Electrocardiogram
ECs: Endothelial cells
FBS: Fast blood sugar
FDA: Food and Drug Administration
GMP: Good manufacturing practice
HGF: Hepatocyte growth factor
HLI: Hind limb ischemia
IA: Intra-arterial
IL: Interleukin
IM: Intramuscular
INF-γ: Interferon gamma
ISCT: International Society of Cell Therapy
KJF: Keratinocyte growth factor
MHC: Major histocompatibility complex
MRA: Magnetic resonance angiography
MSC: Mesenchymal stem cells
MTD: Maximum tolerable dose
NA: Not applicable
NK: Natural killer cells
PAD: Peripheral arterial disease
TAO: Thromboangiitis obliterans
TGF-β: Transforming growth factor beta
TNF-α: Tumor necrosis factor alpha
T cell: T lymphocyte
UCB-MSCs: UCB-derived mesenchymal stem cells
VAS: Visual analog scale
VascuQoL-6: Vascular quality of life questionnaire-6
VEGF: Vascular endothelial growth factor
