# Supplementary material for: Report of a phase 1 clinical trial for safety assessment of human placental mesenchymal stem cells therapy in patients with critical limb ischemia (CLI)
Source: Stem Cell Res Ther. 2023 Jul 5;14:174. doi: 10.1186/s13287-023-03390-9 (PMC10324209; doi:10.1186/s13287-023-03390-9)
Supplement: Supplementary file 1 — Additional file 1: Figure S1 Flow cytometric characterization of PM-MSCs. The cells were negative for CD45, CD34. They displayed positive expression for MSCs markers; CD105, CD90, and CD29. Figure S1.a Placenta 1. Figure S1.b Placenta 2. Figure S2 Flow cytometry of CD4 and CD8 and CD25 on patients with CLI. a Patient 2, A. CD4/CD25 before P-MSC injection, B. CD4/CD25 after P-MSC injection, C. CD4/CD8 before P-MSC injection, D. CD4/CD8 after P-MSC injection. b. Patient 4, A. CD4/CD25 before P-MSC injection, B. CD4/CD25 after P-MSC injection, C. CD4/CD8 before P-MSC injection, D. CD4/CD8 after P-MSC injection. c. Patient 9, A. CD4/CD25 before P-MSC injection, B. CD4/CD25 after P-MSC injection, C. CD4/CD8 before P-MSC injection, D. CD4/CD8 after P-MSC injection. Table S1 Comparison of CD4, CD8, and CD25 markers results on baseline and 6 months after implantation of P-MSCs. Table S2 Laboratory findings before the first and after the last cell infusions. Table S3 Characteristics of placenta donors. [file 13287_2023_3390_MOESM1_ESM.docx]

Supplementary data file for:

**Report of a phase 1 clinical trial for safety assessment of human placental mesenchymal stem cells therapy in patients with Critical limb ischemia (CLI)**

Zeinab Shirbaghaee^1^, Saeed Heidari Keshel^1^, Mehdi Rasouli^1^, Majid Valizadeh^2^, Seyed Saeed Hashemi Nazari^3^, Mohammad Hassani^4^*, and Masoud Soleimani^1,5^ *

^1^Department of Tissue Engineering and Applied Cell Sciences, School of Advanced Technologies in Medicine, Shahid Beheshti University of Medical Sciences, Tehran, Iran.

^2^Obesity Research Center, Research Institute for Endocrine Sciences, Shahid Beheshti University of Medical Sciences

^3^Prevention of Cardiovascular Disease Research Center, Department of Epidemiology, School of Public Health and Safety, Shahid Beheshti University of Medical Sciences, Tehran, Iran.

^4^Department of Vascular and Endovascular Surgery, Ayatollah Taleghani Hospital Research Development committee, Shahid Beheshti University of Medical Sciences, Tehran, Iran.

^5^Applied Cell Sciences and Hematology Department, Faculty of Medical Sciences, Tarbiat Modares University, Tehran, Iran.

**Corresponding authors**

**Dr. Masoud Soleimani.**

E-mail: [Soleim_m@modares.ac.ir](mailto:Soleim_m@modares.ac.ir)

**Dr. Mohammad Hassani**

E-mail: [drmhasani57@sbmu.ac.ir](mailto:drmhasani57@sbmu.ac.ir)

**Supplementary data file include Fig S1 to S2 and Table S1 to S3**

**Figure S1.** Flow cytometric characterization of PM-MSCs. The cells were negative for CD45, CD34 (hematopoietic stem cell markers). They displayed positive expression for MSCs markers; CD105, CD90 and CD29.

**Figure S1. a.** Placenta 1
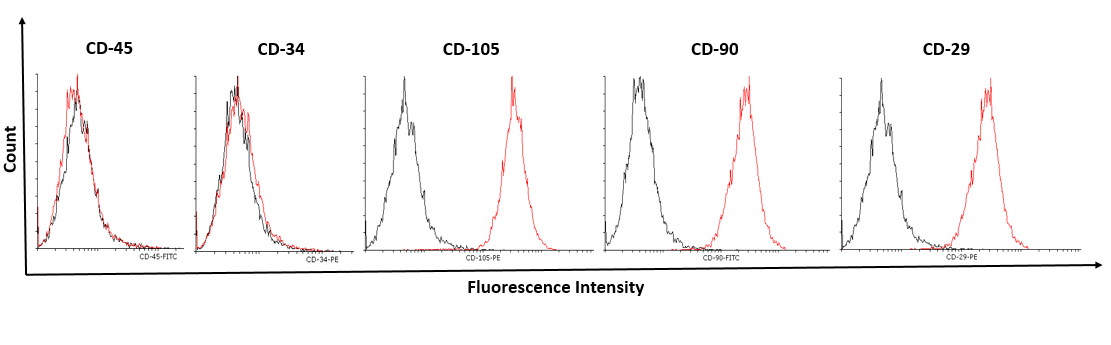


**Figure S1. b.** Placenta 2
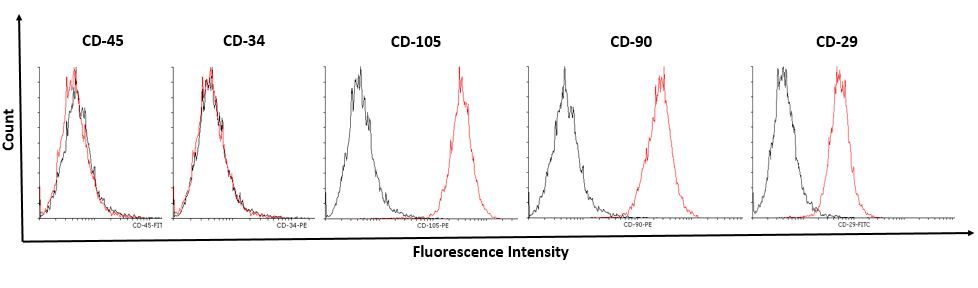


**Figure S2.** Flow cytometry of CD4 and CD8 and CD25 on patients with CLI.


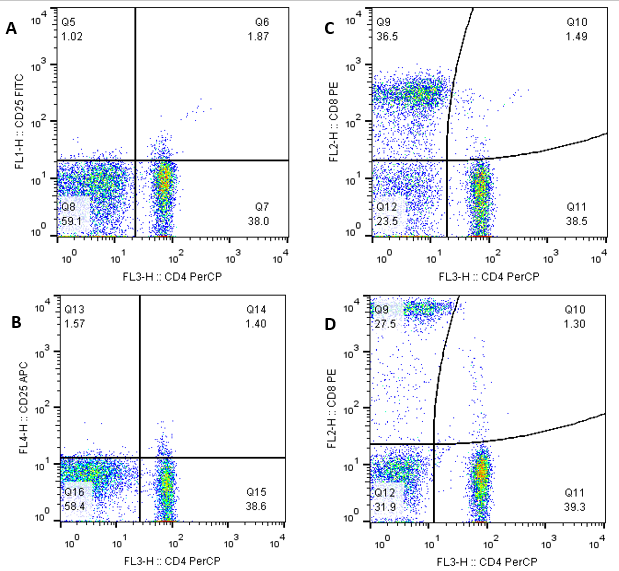


**Figure S2. a. Patient 2**, A. CD4/CD25 before P-MSC injection, B. CD4/CD25 after P-MSC injection, C. CD4/CD8 before P-MSC injection, D. CD4/CD8 after P-MSC injection.


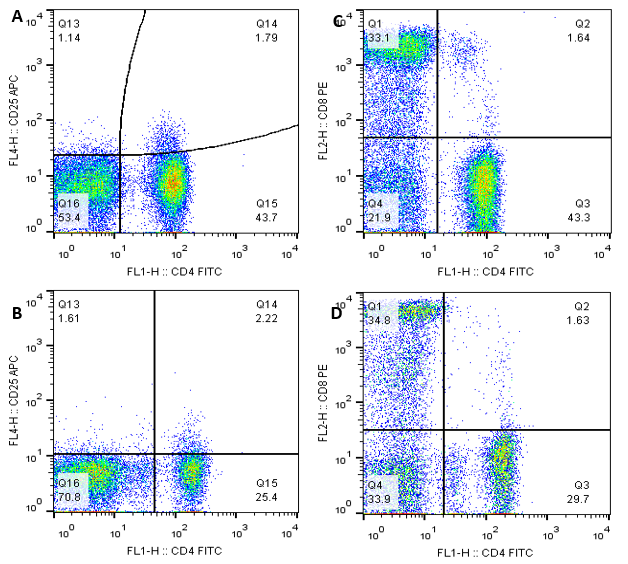


**Figure S2. b. Patient 4**, A. CD4/CD25 before P-MSC injection, B. CD4/CD25 after P-MSC injection, C. CD4/CD8 before P-MSC injection, D. CD4/CD8 after P-MSC injection.


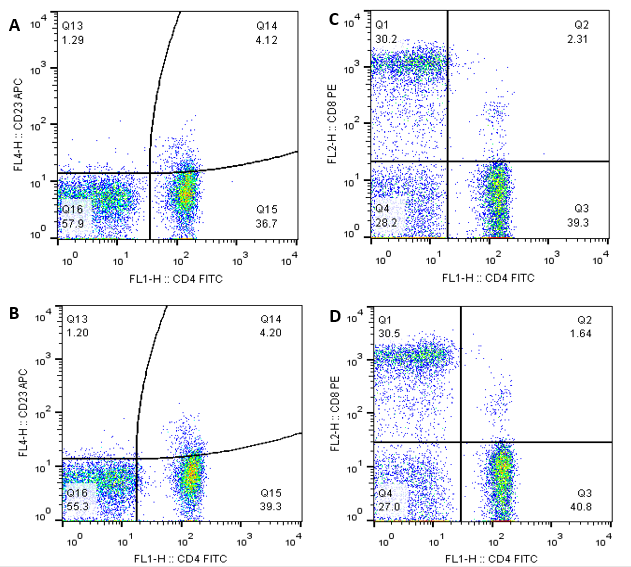


**Figure S2. c. Patient 9**, A. CD4/CD25 before P-MSC injection, B. CD4/CD25 after P-MSC injection, C. CD4/CD8 before P-MSC injection, D. CD4/CD8 after P-MSC injection.

**Table S1.** Comparison of CD4, CD8, and CD25 markers results on baseline and 6 Months after implantation of P-MSCs

| Case number | CD4% | | CD8% | | CD25% | | CD4/CD25% | | CD4/CD8% | |
| --- | --- | --- | --- | --- | --- | --- | --- | --- | --- | --- |
|  | Before | After | Before | After | Before | After | Before | After | Before | After |
| 1 | 46.1 | 50.8 | 21.1 | 22.9 | 1.3 | 1.46 | 4.98 | 2.7 | 0.43 | 9.03 |
| 2 | 38.5 | 39.3 | 36.5 | 27.5 | 1.02 | 1.57 | 1.87 | 1.4 | 1.49 | 1.3 |
| 3 | 43.1 | 45.5 | 22.9 | 22 | 1.61 | 0.54 | 3.75 | 1.86 | 7.53 | 4.76 |
| 4 | 43.3 | 29.7 | 33.1 | 34.8 | 1.14 | 1.61 | 1.79 | 2.22 | 1.64 | 1.63 |
| 6 | 44.1 | 20.2 | 31.7 | 18 | 3.17 | 1 | 8.61 | 2.77 | 2.99 | 1.09 |
| 7 | 42.2 | 44.8 | 40.9 | 39.3 | 0.77 | 0.74 | 3.32 | 4.18 | 3.13 | 2.96 |
| 8 | 45.1 | 45.6 | 31.4 | 20.5 | 1.09 | 1.64 | 3.84 | 5.31 | 1.83 | 0.32 |
| 9 | 39.3 | 40.8 | 30.2 | 30.5 | 1.29 | 1.21 | 4.12 | 4.2 | 2.31 | 1.64 |
| mean(SD) | 40.8 | 33.23 | 28.33 | 24.07 | 1.26 | 1.03 | 3.41 | 2.74 | 2.61 | 1.71 |

**Table S2**. Laboratory findings before the first and after the last cell infusions.

| Variables | **Patient 1**  Base line, 6month | **Patient 2**  Base line, 6month | **Patient 3**  Base line, 6month Base line, , 6 month | **Patient 4**  Base line, 6month | **Patient 5** | **Patient 6**  Base line, 6month | **Patient 7**  Base line, 6month | **Patient 8**  Base line, 6month | **Patient 9**  Base line, 6month | **Normal Range** | **Mean (SD)**  Base line, 6month | **Significantly different (P ˂ 0.05)?** |
| --- | --- | --- | --- | --- | --- | --- | --- | --- | --- | --- | --- | --- |
| **WBC count (× 10^9^/L)** | 7800 6500 | 9300 13000 | 11800 13200 | 15300 14600 | 27700 25000 | 10500 11000 | 11700 11500 | 11200 12200 | 10500  11000 | 3400-12500 | 12.87  13.11 | No |
| **Fbs(mg/dl)** | 90 86 | 200 180 | 70 75 | 205 131 | 200 190 | 84 80 | 114 96 | 86 76 | 97 95 | 70-115 | 127.33  112.11 | Yes |
| **Hb A1C (%)** | 5.1 5.2 | 7 6.5 | 5.7 5.3 | 7 6.5 | 7 7 | 5.1 5.2 | 6.5 6 | 5.1 5.1 | 5 4.9 | 3-6 | 5.94  5.74 | No |
| **BUN (mg/dl)** | 16 20 | 11 8 | 11 8 | 11 10 | 15 13 | 10 11 | 14 12 | 13 12 | 11 9 | 8-21 | 12.44  11.44 | No |
| **Cr (mg/dl)** | 0.8 1.3 | 1.1 0.8 | 1.2 0.8 | 1 0.9 | 0.8 1 | 1 0.9 | 1 0.9 | 1.1 1 | 0.8 0.8 | 0.7-1.4 | 0.98  0.93 | No |

**Table S3**. Characteristics of placenta donors

| Donor No | age | birth history | Medical status | COVID-19 vaccine | Hepatitis B Vaccine | Tetanus Vaccines |
| --- | --- | --- | --- | --- | --- | --- |
| 1 | 26 | Yes/ 1 | healthy | BBIBP-CorV( Sinopharm)/ 3 dose | Recombivax HB/ 3 dose | Tdap |
| 2 | 24 | No | healthy | BBIBP-CorV( Sinopharm)/ 3 dose | Recombivax HB/ 3 dose | Tdap |
